# Use of user-centered design to create a smartphone application for patient-reported outcomes in atopic dermatitis

**DOI:** 10.1038/s41746-018-0042-4

**Published:** 2018-08-13

**Authors:** Lia E. Gracey, Shiyi Zan, Joseph Gracz, John J. Miner, Jacqueline F. Moreau, Jodi Sperber, Kamal Jethwani, Timothy M. Hale, Joseph C. Kvedar

**Affiliations:** 1 Department of Dermatology, Massachusetts General Hospital, Boston, MA 02114 USA; 2 Partners HealthCare Connected Health, Boston, MA 02114 USA

**Keywords:** Quality of life, Outcomes research

## Abstract

The ubiquity and convenience of smartphones carries great potential for collecting patient-reported data to address many gaps in research, especially those that rely on ongoing, real-time data collection. Health care apps have often suffered from low utility due to lack of consideration of the needs of multiple stakeholders. We employed an iterative user-centered design approach to create the myEczema smartphone application (app) to study the burden of disease of atopic dermatitis. We outline below the steps we took for developing myEczema for multiple stakeholders, including patients, clinicians, and researchers.

## Introduction

Atopic dermatitis, often called “eczema”, is the most common inflammatory, chronic skin disease of children.^1^ The etiology of eczema is still incompletely understood, but is thought to have a strong genetic component leading to an impaired skin barrier, with likely contributions from weather conditions and environmental irritants.^2^ While it is known that eczema can have a profound impact on the quality of life of patients and their families,^3^ it has been difficult for clinicians and researchers to track long-term outcomes, follow symptoms on a more granular level, and track affordability of eczema treatments.

At the time of the project, there were 12 eczema smartphone apps on iTunes, with most focusing on tracking eczema “triggers” and treatments, and none that were focused on collecting user data for research purposes. In our clinical experience, patients did not regularly use smartphone apps to manage their eczema either due to lack of knowledge of their existence or lack of usefulness. Clearly, there was a disconnect and lack of value that was being provided from eczema tracking apps. We used design thinking to create myEczema given its roots in human-centered design for “ordinary people” and the use of multidisciplinary teams that are trying to achieve a desirable product that is empathetic to the needs of its users.^4^

## Results

### Focus group and clinician interview findings

From the focus groups, we found that the majority of people already tracked their own or their child’s skin condition in a variety of methods, such as using Google calendar, handwritten journals, or taking photos of their skin that were mixed in with all of their other photos. We were fascinated to find that despite the fact that most patients with eczema already had tracking behaviors for triggers, there was still a missing link that kept them from using other pre-existing eczema apps, even those that provided digital tracking of triggers. In trying to bridge this gap, we found several themes that emerged from the focus groups:

a. A desire to know what external triggers were leading to eczema flares.

b. A way to organize photos and freehand thoughts about their (or their child’s) eczema.

c. A display feature with feedback on their eczema activity.

d. An altruistic desire to contribute to eczema research to move toward a cure such that people in the future would not suffer from the disease.

e. An immediate pipeline for reaching their physician.

f. A way to improve their itch, loss of sleep, and appearance of their skin.

Notably, the most significantly different pain point for parents of children with eczema compared to adult patients was difficulty in getting their children to cooperate with the eczema treatments (usually topicals) which can sometimes be greasy and require sitting still. Several parents requested a game feature to get their children to be more invested in their care. We had initially wanted to include a game feature but unfortunately it did not fit in the budget for development. We also had feedback from several caregivers who reported a workaround of letting young children have alternative screen time with videos on their smartphones while applying the topicals.

Interestingly, our interviews with clinicians highlighted the danger of having patients obsessively focus on tracking external behaviors, such as diet, and we did not want to feed into this behavior. Patients and families often feel frustrated when they think there must be an external trigger they are missing when their eczema is active; however, there is often an intrinsic genetic component to eczema that cannot be modified. Instead of focusing on tracking triggers, several dermatologists suggested to follow symptoms of itch. The majority of clinicians thought that itch was the most interesting symptom, as many feel that severe itch ultimately drives poor sleep, difficulty paying attention at school/work, and breakdown of the skin barrier from scratching. It was universally agreed upon that any tool that a caregiver or patient brought to a clinic visit would have to provide instant, easy-to-interpret information, ideally in a calendar format that would provide a birds-eye view. It can often be a challenge for adult patients and caregivers of children with eczema to accurately articulate how their skin has been doing over the last few months during a short appointment, especially if their skin is inactive the day of the visit.

Based on the background research from patient focus groups and physician interviews, we found common ground for patients and dermatologists: both groups have a focus on symptoms of itch as driving downstream problems, both thought a birds-eye view of symptom severity could be useful, and all agreed that supporting further eczema research using patient-reported outcomes was paramount. The main sources of discord were patients wanting ways to immediately get in touch with their clinician (via phone call or appointments) and focusing on figuring out dietary or other external causes of eczema, which were neither necessarily feasible nor supported by clinicians. The rise of telemedicine has made immediate contact with a clinician possible, but we chose not to add this pipeline given concerns that this could lead to fragmentation of care as most platforms were not easily integrating with all EMRs at the time of this project.

### Features of myEczema

**Fig. 1:**
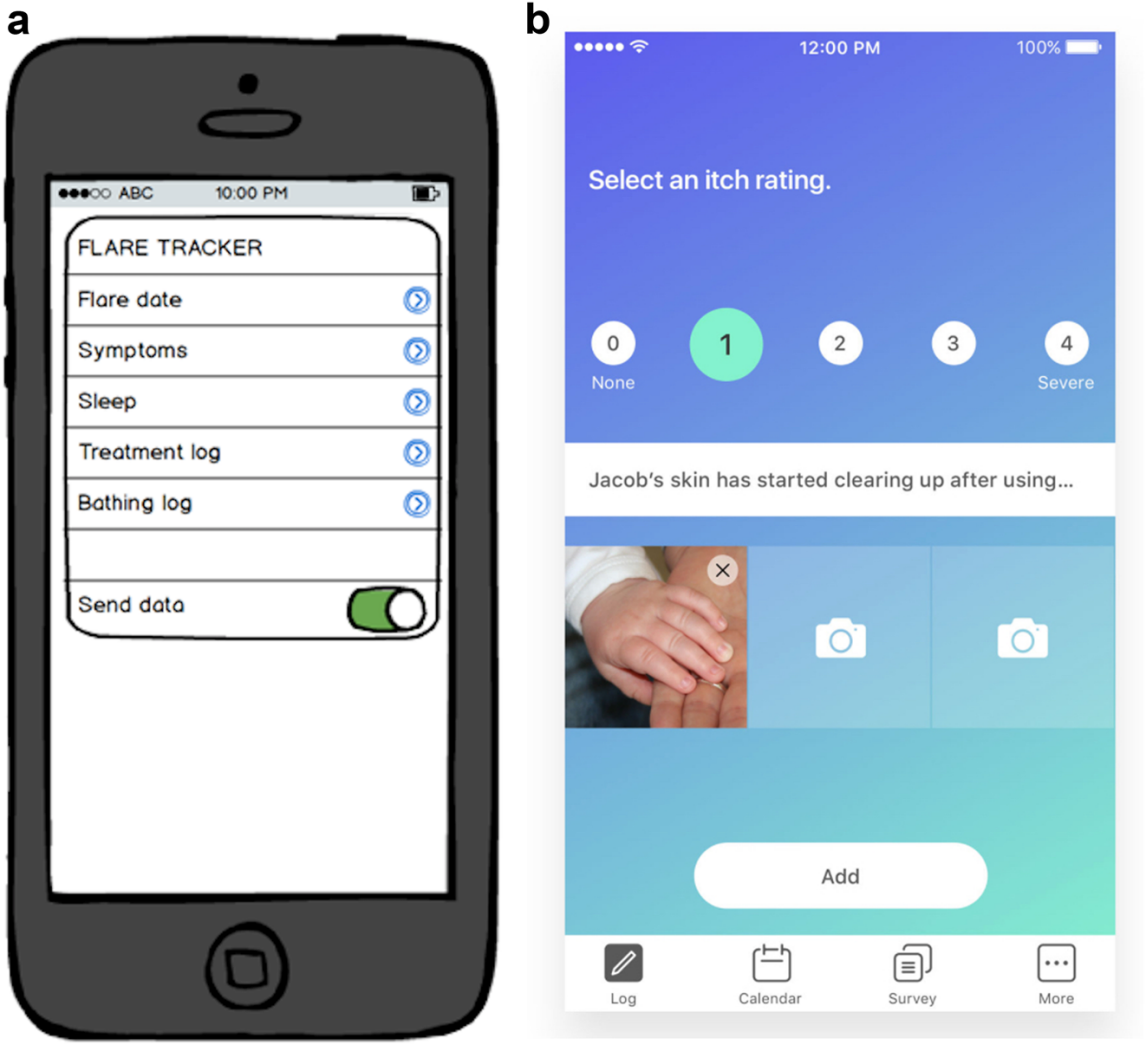
Evolution of the design of myEczema. **a** Initial prototype. **b** Final product based on input and feedback from focus groups and dermatologists
- Calendar View—Quickly visualize Itch Log entries with associated photos and see a birds-eye view of eczema activity over time to facilitate communication during a doctor’s visit.
- Brief surveys—Short intermittent surveys were included to allow the authors to study patient demographics, quality of life metrics, and treatments used for burden of disease research. The surveys were no longer than 10 questions, requiring less than 2 min to complete. In promotion of the myEczema app, we aimed to send a clear message that the purpose of app is to facilitate participating in eczema research and does not take the place of seeking care from a physician.

Given these insights, we decided upon the following core features:

- Itch Log feature (Fig. 1)—A user can quickly select an itch severity rating and add photos and notes to stay organized. We focused on one interaction that only takes a few seconds to complete and could provide useful longitudinal research data points.

One of the most significant distinctions between the myEczema app and the other eczema apps on the market was our emphasis on directly contributing to eczema research. Importantly, the development of myEczema was led by a clinician-researcher. Eczema is a condition with a high emotional investment from patients and their families. We were struck by the inspiring altruism of eczema sufferers and caregivers to help others deal with the burden of this disease in the future. Several of the other eczema apps on the market asked users to input data about multiple symptoms but this data was dead-end in the app and would not be used for the greater good of research. Over half of the clinicians we interviewed were also eczema researchers. We largely chose which survey questions to ask, which metrics to use, and ultimately focused on itch as the primary symptom to measure based on the feedback of thought leaders in eczema research.

The myEczema app also uniquely promoted and linked to a community support site (Eczema Wise hosted on Inspire), where patients and caregivers could connect with each other, share their feelings, and swap advice.

Several of the other eczema apps on the market would give feedback to users in the form of a line graph that illustrates severity or frequency of eczema flares. The authors felt this display was less useful, and sometimes misleading, especially if a great amount of time happened in between entries. We instead chose a monthly calendar format where a user can quickly see the days where they had entries, with a colored dot signifying severity of itch and the photos/notes from that day.

One of the behaviors we observed from eczema patients was the ubiquity of taking photos of their skin with their smartphones. These photos were usually mixed in with tens to hundreds of other photos of their family, pets, etc., making it difficult to find “that one photo” that they wanted to show their doctor during their time-pressed appointment. myEczema provides a streamlined design with a quick way to tie a photo to an itch rating and any freehand notes that a user wants to add. This is then displayed in the calendar feature for quick reference. None of the other apps on the market focused on itch on the home screen and only one other eczema app had a straightforward way of adding photos to a daily entry.

### Testing and data collection

For testing the prototype, we put screenshots in front of another focus group to ensure the UI was intuitive for the Itch Log home screen feature. We also asked users about the length of questions they could tolerate on a smartphone app. The consensus was that the Itch Log rating was feasible at least once a week since it was such a quick interaction. The longer 10-question validated quality of life surveys were thought to be attainable once a month.

From a research perspective, we needed a secure database that would be readily accessible and updated in real time without high costs. We were able to achieve this using Amazon Web Services. New data is updated on the server daily at a monthly cost of less than $2. We used randomly generated patient ID numbers and only collected itch ratings and survey responses about demographics, quality of life, and treatment surveys. We did not collect and store photos or freetext notes that were entered.

Preliminary data collection from August 2017 to January 2018 showed average usage statistics shown in Table 1. The Itch Log and Calendar View appeared to be the most popular features. This was expected as the Itch Log is the home screen when users return into the app. The itch data was the priority for us to collect to follow on a longitudinal basis, thus we wanted to feature it on the home screen with a design that would allow entry in a matter of seconds. The Calendar View was anticipated to be the most useful tool for the user as this was intended to help communicate how the patient’s eczema had been doing with quick links to notes, photos, and itch ratings. The survey tab led to the 10-question survey and unsurprisingly was the least used given that it was the biggest ask and time commitment for the user.

**Table 1:** Average number of times that myEczema users interacted with each feature per month

	Feature			
	Itch Log	Calendar View	Surveys	More^a^
Average instances per month	326.5	386.8	59.5	110.3

^a^The “More” feature included Treatment Surveys, Notification settings, links to the National Eczema Association and Eczema Wise support forum websites, About, and Terms of Service

## Discussion

Data collection is currently ongoing with users from the United States, Canada, United Kingdom, Australia, and Singapore. Notably, we did minimal promotion for the myEczema smartphone app and mostly relied on social media channels via the National Eczema Association, which focuses on patients with eczema in the United States. Overall, we aimed to design myEczema to provide a streamlined way for patients with eczema to participate in meaningful research while giving users a tool for logging and displaying eczema activity to facilitate the care they receive from clinicians.

## Methods

As a first step in the design process, we conducted focus groups to uncover “pain points” and features that patients would want from an eczema app. The first focus group was conducted at Boston Children’s Hospital with parents of children with eczema before we started development of the app. An additional three focus groups were held approximately 4 months into the design process, during a National Eczema Association educational forum in San Francisco, CA. These focus groups were a roughly 50-50 split of adult patients and caregivers of children with eczema, with each group consisting of 15–20 people. A final focus group was held after the final prototype was created to get feedback from adult patients and caregivers of children with eczema.

Although myEczema was intended to be a patient-facing app, the project was led by a clinician and we wanted to get input on useful features from clinicians as stakeholders. We spoke with eight dermatologists in academic and private practice settings across the United States and asked open-ended questions about what data they would find interesting, what symptoms were important to measure, and how they would envision themselves utilizing a patient-focused eczema app during a clinic visit (if at all). The majority of the clinicians were also eczema researchers.

The myEczema smartphone application was listed on iTunes as a free download in July 2017.^5^ Mixpanel freeware was used to track usage statistics. The secondary data analysis was approved by the Partners HealthCare Institutional Review Board. Users had to accept the terms and conditions prior to using the application.

### Data availability

The data that support the findings of this study are available from the corresponding author upon reasonable request.

### Code availability

The custom code used to generate the myEczema smartphone application will be available upon reasonable request to the corresponding author.
